# Increasing Inhibition of the Rat Brain 2-Oxoglutarate Dehydrogenase Decreases Glutathione Redox State, Elevating Anxiety and Perturbing Stress Adaptation

**DOI:** 10.3390/ph15020182

**Published:** 2022-01-31

**Authors:** Artem V. Artiukhov, Anastasia V. Graf, Alexey V. Kazantsev, Alexandra I. Boyko, Vasily A. Aleshin, Alexander L. Ksenofontov, Victoria I. Bunik

**Affiliations:** 1 Department of Biokinetics, A. N. Belozersky Institute of Physico-Chemical Biology, Lomonosov Moscow State University, 119234 Moscow, Russia; whitelord32br@gmail.com (A.V.A.); nastjushka@gmail.com (A.V.G.); mak@org.chem.msu.ru (A.V.K.); aleshin_vasily@mail.ru (V.A.A.); ksenofon@belozersky.msu.ru (A.L.K.); 2 Department of Biochemistry, Sechenov University, 105043 Moscow, Russia; 3 Faculty of Biology, Lomonosov Moscow State University, 119234 Moscow, Russia; 4 Faculty of Chemistry, Lomonosov Moscow State University, 119234 Moscow, Russia; 5 Faculty of Bioengineering and Bioinformatics, Lomonosov Moscow State University, 119234 Moscow, Russia; boiko.sash@gmail.com

**Keywords:** succinyl phosphonate, triethyl succinyl phosphonate, 2-oxoglutarate dehydrogenase, redox potential, anxiety, locomotion

## Abstract

Specific inhibitors of mitochondrial 2-oxoglutarate dehydrogenase (OGDH) are administered to animals to model the downregulation of the enzyme as observed in neurodegenerative diseases. Comparison of the effects of succinyl phosphonate (SP, 0.02 mmol/kg) and its uncharged precursor, triethyl succinyl phosphonate (TESP, 0.02 and 0.1 mmol/kg) reveals a biphasic response of the rat brain metabolism and physiology to increasing perturbation of OGDH function. At the low (TE)SP dose, glutamate, NAD^+^, and the activities of dehydrogenases of 2-oxoglutarate and malate increase, followed by their decreases at the high TESP dose. The complementary changes, i.e., an initial decrease followed by growth, are demonstrated by activities of pyruvate dehydrogenase and glutamine synthetase, and levels of oxidized glutathione and citrulline. While most of these indicators return to control levels at the high TESP dose, OGDH activity decreases and oxidized glutathione increases, compared to their control values. The first phase of metabolic perturbations does not cause significant physiological changes, but in the second phase, the ECG parameters and behavior reveal decreased adaptability and increased anxiety. Thus, lower levels of OGDH inhibition are compensated by the rearranged metabolic network, while the increased levels induce a metabolic switch to a lower redox state of the brain, associated with elevated stress of the animals.

## 1. Introduction

The pathogenic significance of impaired brain metabolism is acquiring increasing attention. In particular, mitochondrial dysfunction is known to mediate the most challenging diseases of the central nervous system, such as neurodegenerative [1,2,3] and psychiatric [4] disorders. Metabolic impairments observed in these pathologies usually involve the deficient function of mitochondrial proteins, elevated oxidative and nitrosative stress, decreased oxidative phosphorylation, and altered Ca^2+^ homeostasis [5,6,7,8,9,10,11]. Altogether, these changes form a vicious cycle ultimately causing neuronal death and a general decline in the physiological fitness of the organism.

Neurological disorders caused by different molecular events may have similar manifestations. Therefore, therapies focused on the symptoms without knowing their causes are far from optimal. The therapeutic correction of impaired brain functions is often based on the regulation of neurotransmission by targeting receptor systems, ion channels, or neurotransmitter recycling systems [11]. Metabolic syndrome is known to be a side effect of such treatments [12,13,14,15], stressing not only the interplay between brain metabolism and neurotransmission but also the potential of metabolic regulation, especially in brain mitochondria, to treat neuronal pathologies. 

A key mitochondrial reaction often perturbed in brain diseases is a rate-limiting step of the mitochondrial TCA cycle, catalysed by 2-oxoglutarate dehydrogenase (OGDH) [16]. OGDH, also known as the E1 component of the 2-oxoglutarate dehydrogenase complex (OGDHC), is of utmost importance not only for energy production but also for de novo generation of the glutamate neurotransmitter from glucose in the TCA cycle. Indeed, the level of glutamate, the main excitatory neurotransmitter in the central nervous system, is tightly linked to the brain’s OGDHC function [17]. This link may underlie different physiological outcomes of the OGDH-directed regulation in healthy organisms [18,19] and those with neuropathologies [20,21,22,23], as well as natural variations in the activity of this mitochondrial complex in different brain regions and physiological settings [17,18]. Pathogenic variants of OGDH and its partners within the multienzyme complex cause developmental delay, elevated lactate, ataxia, and seizures [24,25,26,27]. While a loss of OGDHC function due to mutations causes early developmental lethality [24,28,29,30], pharmacological regulation of the enzyme helps understand the physiological impact of OGDHC impairment in the adult brain.

**Figure 1:**
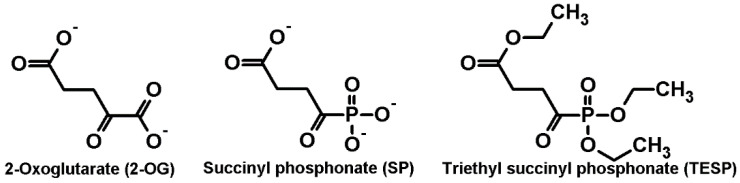
Structures of the OGDH 2-oxo ligands. 2-Oxoglutarate (2-OG), a substrate, and its phosphonate analogues in the anionic and esterified forms inhibiting OGDH in vitro (SP) and in vivo (SP, TESP), are shown.

The goal of the current work is to model neurodegenerative diseases characterized by acquired insufficiency of the cerebral OGDH function [23,31,32,33,34,35,36,37,38,39], using specific inhibitors of the enzyme (Figure 1): a synthetic analogue of 2-oxoglutarate (2-OG), succinyl phosphonate (SP), and its uncharged precursor, triethyl succinyl phosphonate (TESP). Compared to the negatively charged SP, its esterified precursor TESP should have a higher membrane permeability. Inside the cell, SP is produced from TESP through intracellular de-esterification [40,41].

The OGDHC inhibitors and the range of their doses studied in this work take into account our previous experience regarding specificity and efficiency of the action of the phosphonate analogs of 2-oxoglutarate in different eukaryotic systems [42,43,44]. In particular, in various rat models of pathologies, the dose dependence of physiological responses may be observed between 0.02 and 0.1 mmol of SP per kg [19,45]. Moreover, esterified 2-oxo phosphonates show a steeper dose-response dependence in cellular studies, compared to the charged precursors [46,47].

In view of the tight link of metabolic dysfunctions, perturbed neurotransmission, and neurodegeneration, understanding the brain reactivity to increasing levels of perturbation in the OGDH-dependent key point of mitochondrial metabolism, gained in the present work, may help the development of new treatments to fight neuropathologies.

## 2. Results

### 2.1. Biphasic Changes in Biochemical Parameters of the Rat Cerebral Cortex in Response to Increasing Dosage of 2-Oxo Phosphonate Inhibitors of OGDH

**Figure 2:**
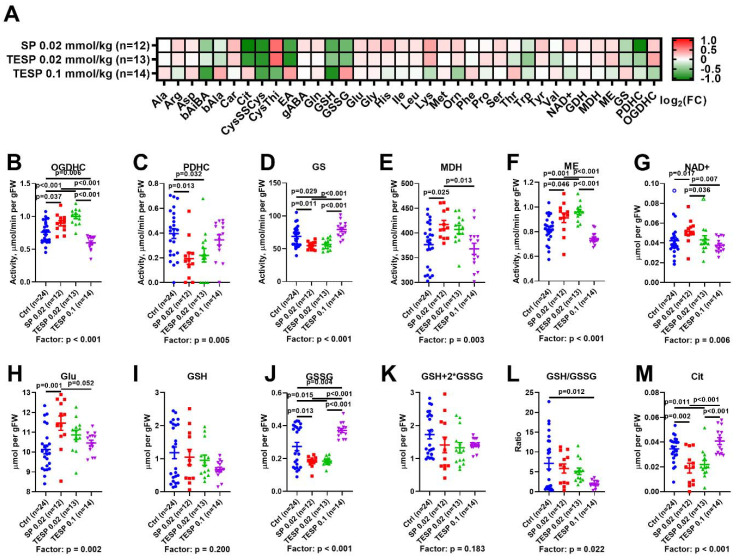
Effects of the succinyl phosphonate (SP) and its triethyl ester (TESP) on the biochemical indicators in the rat cerebral cortex. (**A**) Levels of amino acids and related compounds, together with enzyme activities, presented as log2 of the fold change from the average of the non-treated animals. (**B**–**F**) Levels of 2-oxoglutarate dehydrogenase complex (OGDHC, **B**), pyruvate dehydrogenase complex (PDHC, **C**), glutamine synthetase (GS, **D**), malate dehydrogenase (MDH, **E**), and NADP^+^-dependent malic enzyme (ME, **F**) activities, respectively, presented as µmol/min per gram of tissue fresh weight (gFW). (**G**–**M**) Levels of glutamate (**G**), NAD^+^ (**H**), reduced glutathione (GSH, **I**), oxidized glutathione (GSSG, **J**), total glutathione (**K**), the ratio between the reduced and oxidized forms of glutathione (**L**), and citrulline (Cit, **M**), presented as µmol per gFW. Significant (*p* ≤ 0.05) differences between experimental groups, estimated by ANOVA with Tukey’s post-hoc test, are indicated on the graphs. The ANOVA *p*-values for the treatment factor significance are shown below the graphs. Hollow points correspond to outliers excluded according to the iterative Grubb’s test. The number of animals in experimental groups (*n*) is indicated including outliers. Proteinogenic amino acids are abbreviated according to the standard 3-letter code. Other abbreviations used are: bAiBA—β-aminoisobutyrate, bAla—β-alanine, Car—carnosine, CysSSCys—cystine, CysThi—cystathionine, EA—ethanolamine, gABA—γ-aminobutyrate, Orn—ornithine, GDH—glutamate dehydrogenase.

The heat map presented in Figure 2A reveals similar biochemical changes in the rat brain after administration of SP and TESP at the low dose of 0.02 mmol/kg, with the changes alleviated after increasing the TESP dose to 0.1 mmol/kg. Statistical analysis of the selected biochemical changes in the four animal groups (Figure 2B–K) confirms the biphasic response of the rat brain biochemical parameters to increasing the dosage of the OGDH inhibitors. At the low dosage, the effects of SP and TESP are similar. Assayed in vitro, the levels of OGDHC activity in the rat cerebral cortex increase significantly upon the administration of the OGDH-directed inhibitors at 0.02 mmol/kg (Figure 2B). As shown earlier, this increase manifests compensatory activation of the enzyme in response to its inhibition in vivo [19]. The associated changes in the OGDHC-linked enzymes include decreased activities of PDHC (Figure 2C) and glutamine synthetase (Figure 2D) and increased activities of malate dehydrogenase (Figure 2E) and NADP^+^-dependent malic enzyme (Figure 2F). An increase in the TESP dosage from 0.02 to 0.1 mmol/kg results in the abrogation of most of the effects on the rat brain biochemistry, observed at the lower dosage (Figure 2). Except for OGDHC activity, all the enzymatic activities return to control levels.

The complex effects of SP and TESP on the dehydrogenases are reflected in the brain levels of NAD^+^ (Figure 2G) and glutamate (Figure 2H), demonstrating biphasic changes along with the enzymatic activities. Simultaneously, biphasic changes in the levels of redox-active compounds are observed (Figure 2I−M), including the components of glutathione redox buffer and a marker of ^•^NO production, citrulline [48,49].

Thus, the response of the rat brain metabolism to the OGDH inhibitors is biphasic: The enzymatic activities and metabolites, affected at the lower inhibitor dosage, mostly return to the control values upon increasing the dosage. However, some parameters do not return to the control values, but show opposite changes regarding the respective control values at the low and high levels of OGDH inhibition. That is, the low dosage of inhibitors elevates OGDHC activity, decreasing the level of oxidized glutathione. In contrast, the high TESP dosage decreases the OGDHC activity, increasing the level of oxidized glutathione (Figure 2B,J). These two indicators point to a new state of the brain metabolic network, established at the high level of OGDH inhibition. This new state is characterized by decreased redox potential, obvious from the decreased ratio of the reduced to oxidized glutathione (Figure 2L). At the similar content of total glutathione (Figure 2K), the level of glutathione disulphide is significantly increased (Figure 2J) in the brain with strongly inhibited OGDH. 

### 2.2. Effects of OGDH Inhibition on the ECG Parameters and Animal Behavior

**Figure 3:**
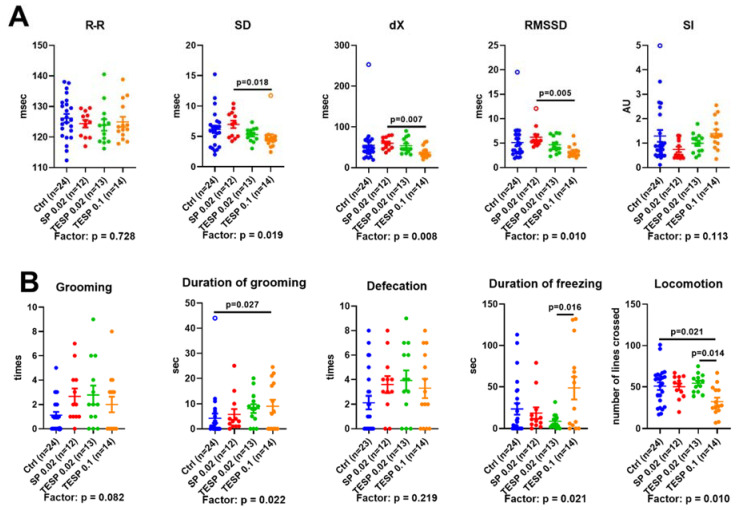
The effects of the succinyl phosphonate (SP) and its triethyl ester (TESP) on the ECG (**A**) and anxiety (**B**) parameters. (**A**) Animals were subjected to the non-invasive ECG to determine the heart rate (R-R interval) and its variability parameters: standard deviation (SD), range (dX), root mean square of successive differences (RMSSD), and stress-index (SI). (**B**) Levels of anxiety indicators and locomotion in the “Open Field” test. Significant (*p* ≤ 0.05) differences between experimental groups, estimated by ANOVA with Tukey’s post-hoc test, are indicated on the graphs. The ANOVA *p*-values for the treatment factor significance are shown below the graphs. Hollow points correspond to outliers excluded according to the iterative Grubb’s test. The number of animals in experimental groups (*n*) is indicated including outliers. AU—arbitrary units.

As shown in Figure 3A, significant differences in the ECG parameters are observed only between the animals treated with 0.02 mmol/kg SP and those treated with 0.1 mmol/kg TESP. This finding implies biphasic changes in the ECG parameters: The minor changes at a low level of the OGDH inhibition are opposite to those at a high level of the inhibition. As a result, the statistically significant difference between the ECG parameters of the animals with the low and high levels of OGDH inhibition is observed, although each of the treated groups is not significantly different from the control group. Compared to the low dosage group, SD, dX, and RMSSD are significantly reduced, while SI tends to increase (*p* = 0.083) in the high dosage group. Decreased indicators of adaptation (SD, dX, and RMSSD) along with the elevated stress indicator (SI) reveal that, compared to 0.02 mmol/kg (TE)SP, the treatment with 0.1 mmol/kg TESP causes an unfavourable shift in heart regulation, manifesting a general decrease in the stress adaptability of the animals.

In the behavioral Open Field test, the high dosage of TESP causes an increase in the duration of grooming and decreased locomotion vs. the control animals (Figure 3B), indicative of an increase in animal anxiety. Moreover, locomotion and duration of freezing show significant differences between the animals treated with the low and high doses of TESP. Similar to the above considered differences in the ECG parameters, those in behavioral parameters also imply biphasic changes upon the increasing level of OGDH impairment.

### 2.3. Correlations between the Brain Biochemical Markers and Physiological Changes

**Figure 4:**
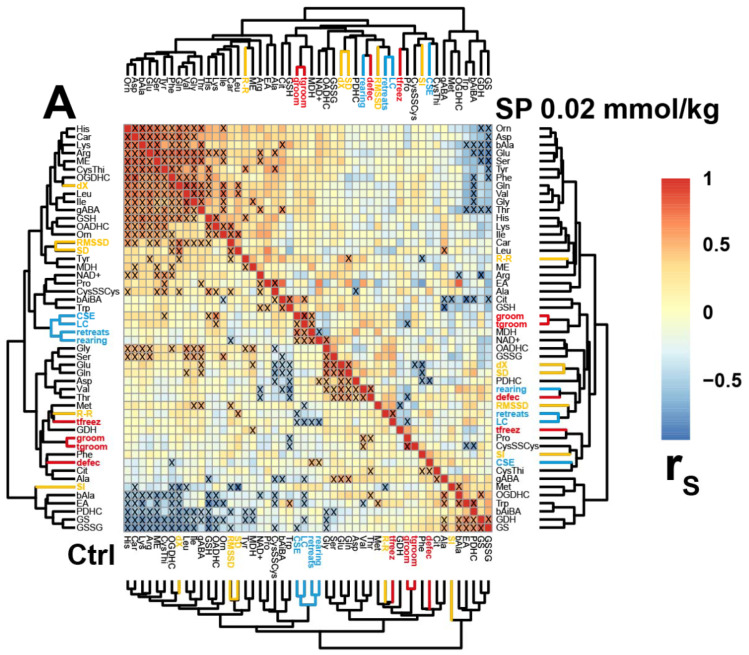

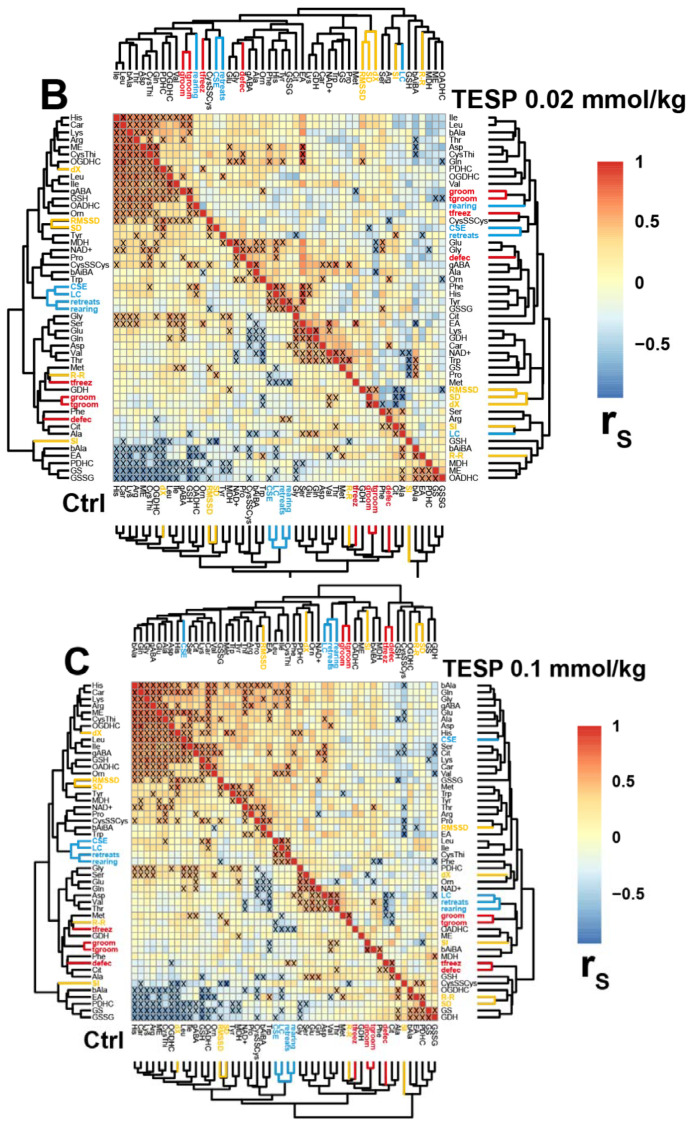
Correlation matrices of amino acid levels and enzyme activities in the brains of rats, subjected to the phosphonates. Heatmaps of Spearman’s correlation coefficients (rS) characterizing the control (Ctrl, *n* = 24, (**A**–**C**)) animals are shown in the bottom left triangles of the figures. In the top right triangles, rS characterizing the animals treated with 0.02 mmol/kg SP (*n* = 12, **A**), 0.02 mmol/kg TESP (*n* = 13, **B**), or 0.1 mmol/kg TESP (*n* = 14, **C**), are shown. Significant (*p* ≤ 0.05) correlations are marked by “x”. Anxiety indicators are written in red, the exploration and locomotion indicators are in blue, the heart rate and its variability are in yellow. Biochemical and physiological parameters are abbreviated as in Figure 2 and Figure 3. Other abbreviations are: groom—number of grooming acts, tgroom—duration of grooming, defec—number of defecation acts, tfreez—duration of freezing, CSE—central square entries, LC—locomotion.

Although correlations between specific biochemical and physiological parameters do not manifest causal relationships, analysis of the correlated parameters and their changes in different (patho)physiological settings may help decipher molecular mechanisms of the network changes [50,51]. The interdependencies of the characteristic parameters of the control and treated animals are compared in Figure 4. It is important to note that the clusters of correlations of physiological parameters strongly differ in the non-treated and treated groups. For instance, in the non-treated animals, correlations of the ECG stress marker SI form a common cluster with the oxidized glutathione (bottom left triangles in Figure 4A–C). In the SP-treated animals, SI is in the cluster with the glutathione precursor cystine (top right triangle in Figure 4A), in the animals treated with a low TESP dose—in the cluster with the ^•^NO precursor arginine (top right triangle in Figure 4B), and in the animals treated with a high TESP dose—in the cluster with the dehydrogenases producing reducing equivalents in the form of NAD(P)H (top right triangle in Figure 4C). Overall, the correlations of the ECG stress index (SI) reveal its coupling to different redox-active metabolites and processes in the brain. The fact that the identity of these metabolites and processes changes dependent on the OGDHC perturbation level suggests that the accompanying metabolic rearrangement may switch essential interdependencies from one component or process of the pathways determining the brain redox state, to another. In particular, in the control animals, the OGDHC activity exhibits strong positive correlations with the brain levels of glutathione and its precursor cystine, as well as a strong negative correlation with the brain level of oxidized glutathione. All these correlations become insignificant in the animals treated with the OGDHC inhibitors. Thus, the correlation analysis reveals that the network supporting the glutathione homeostasis is perturbed already at the low OGDH inhibition levels.

It is worth noting that in the control animals the intervals between the heartbeats (R-R) are clustered together with the anxiety parameters (shown in red in the bottom left triangles in Figure 4A–C), in accordance with the physiological significance of the adaptation of heart rate to a perceived danger. In the animal group treated with a high dosage of the OGDH-directed inhibitor, the anxiety parameters do not form a common cluster, distributed between the cluster including R-R and SD (duration of freezing, defecation) and another one including SI (duration of grooming and number of grooming acts). Thus, the correlation analysis (Figure 4) supports the coupled changes in the parameters of ECG and behavior (Figure 3), pointing to the heightened relationship between the anxiety behavior and the ECG stress marker SI in the animals with strongly inhibited OGDH, compared to the control animals where the anxiety level is mostly linked to R-R intervals.

## 3. Discussion

**Figure 5:**
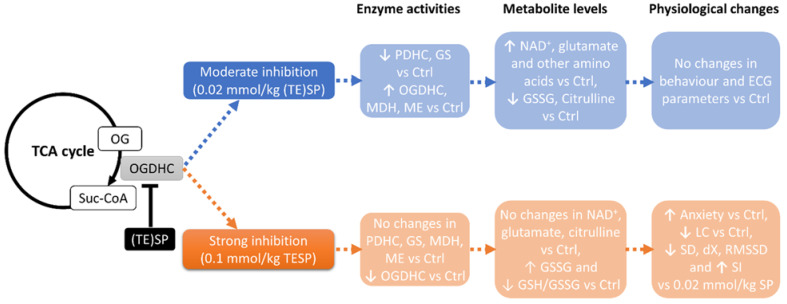
Difference in metabolic and physiological consequences of moderate and strong OGDHC inhibition by (TE)SP in the rat brain. Moderate OGDHC inhibition results in compensatory metabolic rearrangement, which manifests in changed enzymatic activities and metabolite levels, supporting physiological stability. Strong OGDHC inhibition induces a metabolic switch to the brain state with reduced glutathione redox potential (GSH/GSSG), which increases anxiety and decreases adaptability, as revealed by changes in the behavioral and ECG parameters.

Our study has revealed a biphasic response of the brain metabolism to increasing inhibition of OGDHC, which is observed through changes in a number of enzymatic activities and metabolites of the brain. That is, the metabolic changes induced by a low dose (0.02 mmol/kg) of the OGDHC inhibitors (SP or TESP), are abrogated and in some cases reversed with increasing the inhibition (0.1 mmol/kg of TESP) (Figure 5). This feature of the metabolic response to the inhibition of a key mitochondrial process, such as oxidative decarboxylation of 2-oxoglutarate, is inherent in a number of brain enzymatic activities and metabolites, including glutamate, NAD^+^, and citrulline (Figure 2). Although the high level of OGDH inhibition returns many parameters of the system to their initial state, this state differs from the initial one by the decreased levels of the OGDHC activity and glutathione redox potential. Together with the correlation analysis, the data point to the critical role of OGDHC in supporting the brain glutathione biosynthesis, presumably linked to the OGDHC role in the brain metabolism of the glutathione precursors glutamate and cystine [17,52].

The biphasic dose dependence of metabolic effects on OGDH inhibitors in the rat brain (Figure 2) exhibits certain correspondence to the changes in physiological parameters. The second phase, where a switch to a new metabolic state with the decreased OGDHC activity and glutathione redox potential occurs, is translated into increased anxiety, implied by increased duration of freezing and decreased locomotion (Figure 3). The first phase does not correspond to the physiological changes. However, the biphasic physiological changes may be inferred from significant differences between the animals treated with the low and high doses of the OGDH-directed inhibitors. These differences are observed in the ECG parameters, duration of freezing, duration of grooming, and locomotion (Figure 3). Thus, the significant metabolic effects of the low doses of the inhibitors are not translated into physiological changes, while the switch to a new metabolic state of the brain after the high dosage of the OGDH-directed inhibitor does. Our current results on the action of TESP, a membrane-permeable form of SP, are in good accordance with our previous studies on the concentration dependence of SP effects in other animal and cellular models. Regarding the OGDHC activity in the animal brain [19,45] or astrocytes [42], 0.02 mmol/kg SP causes a stronger effect than 0.1 mmol/kg SP. However, while the OGDHC activity decreases with increasing the SP dosage, the physiological effect of SP on the alcohol-induced sleep duration becomes more pronounced [19]. 

Attenuation of the SP metabolic effects upon increasing its dosage also occurred in various cell lines [41,42,43,52,53]. Biphasic concentration dependencies are also known for other drugs [54,55,56]. In particular, increasing doses of antioxidants may result in a reversal of their action to the pro-oxidant one [57,58,59]. 

Overall, our findings, summarized in Figure 5, indicate that the brain possesses a certain capacity to adjust metabolism to a low level of the OGDH inhibition through a compensatory response of the OGDHC-linked metabolic network. This process corresponds to the first phase of the brain metabolic changes, enabling stabilization of the central regulation of ECG and behavior. However, when this response is exhausted, which occurs at the high dosage of the OGDH-directed inhibitor, the brain metabolism is switched to another state. Although this other state is rather similar to the initial one, its redox potential is decreased along with the decreased level of OGDHC activity, which is translated into increased anxiety and decreased capability to adapt the heart rate to metabolic stress.

It is worth noting in this regard that mitochondrial ROS generation upon excitotoxic action of glutamate on hippocampal neurons also shows a biphasic response to SP, decreasing at the low SP dosage and increasing at the higher SP dosage [41]. Biphasic responses of the cellular redox state are also known from independent studies on the cellular OGDH inhibition by carboxyethyl SP or OGDH downregulation by shRNA [60]. As a major system producing ROS in mitochondria under different pathological settings, OGDHC is strongly linked to the glutathione redox buffer through a number of mechanisms, from its post-translational modification by glutathione disulfide to alternative electron flows to the glutaredoxin and thioredoxin systems [61]. Our current work reveals the direct, although complex, link between the brain glutathione redox state and OGDHC function (Figure 2K). The oxidized glutathione level decreases at the low dosage of the OGDH-directed inhibitors, probably reflecting their inhibition of the ROS production in the OGDHC-catalyzed side reactions [61]. However, at the higher dosage of the OGDHC-directed inhibitor, the oxidized glutathione level increases upon metabolic switch to a decreased level of the OGDHC activity, manifesting decreased substrate flux through the TCA cycle. The OGDH-mediated increase in glutathione disulfide is translated into decreased glutathione redox potential in the brain, which increases anxiety and perturbs ECG.

Oxidative disbalance is known to be associated with anxiety [62,63,64], which, in its turn, is often considered an early marker or risk factor for Alzheimer’s disease [65,66,67]. This and other neurodegenerative diseases, characterized by the reduced redox status of the brain, also exhibit the decreased activity of the brain OGDHC, already at the initial study of the disease [68]. Reduced OGDHC function and altered glutathione redox balance are also known in schizophrenia patients [69,70,71]. Manipulating the brain glutathione redox status by specific OGDH-directed inhibitors, we not only have reproduced the increased anxiety in the animal model but demonstrated the causal link between the OGDHC function and glutathione redox state. Our results on the biochemical and physiological effects of the brain OGDHC inhibition unravel molecular mechanisms underlying the known benefits of therapeutic applications of a physiological OGDHC activator, thiamine, and its pharmacological derivatives, in patients with Alzheimer’s disease [72,73], in animal models of neurodegeneration [74,75,76,77], brain and spinal cord injuries [20,21], enhanced stress [78,79,80], and other neurological conditions [81,82,83,84,85,86,87].

## 4. Materials and Methods

### 4.1. Reagents

Succinyl phosphonate (SP) and its triethyl ester (TESP) were synthesized as described previously [40]. NAD^+^ was obtained from Gerbu (Heidelberg, Germany), oxidized glutathione—from Calbiochem (La Jolla, CA, USA). Formate dehydrogenase for NAD+ assays was obtained from the Federal Research Center of Biotechnology/Innotech MSU (Moscow, Russia). All other reagents were of the highest purity available and obtained from Sigma-Aldrich (Helicon, Moscow, Russia).

### 4.2. Animal Experiments

Animal experiments were performed according to the Guide for the Care and Use of Laboratory Animals published by the European Union Directives 86/609/EEC and 2010/63/EU, and were approved by the Bioethics Committee of Lomonosov Moscow State University (protocol number 69-o from 09.06.2016). Wistar male rats were kept at 21 ± 2 °C and a relative humidity of 53 ± 5%, with 4–6 individuals being placed in each cage [88] with the 12/12 h light/dark cycle (lights on 9:00 = ZT 0, lights off 21:00 = ZT 12). Standard rodent pellet food (laboratorkorm.ru) and tap water were available ad libitum. The minimum necessary size of the animal sample was estimated by a *t*-test, as described previously [89]. No rats died or were excluded during the experiment.

SP and TESP were dissolved in physiological solution (0.9% NaCl) to a 0.2 M or 1 M concentration and were administered at a 0.02 mmol/kg or 0.1 mmol/kg dosage, respectively. The administration was conducted intranasally to pass the blood-brain barrier [90,91,92]. The physiological solution was administered to the control animal groups (two groups of 12 animals, total of 24 animals). The treated groups included animals administered with 0.02 mmol/kg SP (12 animals), with 0.02 mmol/kg TESP (13 animals), and with 0.1 mmol/kg TESP (14 animals). Twenty-four hours after the substance administration, the rats were subjected to physiological tests and killed by decapitation as described before [82,89]. Taking into account that anaesthetics influence mitochondrial and brain function [88,93,94,95,96,97,98], rats were killed by decapitation with a guillotine (OpenScience, Moscow, Russia) without anaesthetics. Such a method is in line with the latest recommendations and guidelines [88,97,99]. The brains were excised, cerebral cortices separated on ice, and frozen in liquid nitrogen within 90 s after decapitation. The tissue samples were stored at −70 °C until the biochemical assays.

### 4.3. Physiological Tests

Estimation of behavioral activity was performed in the “Open Field” test (OpenScience, Moscow, Russia) 24 h after the administration of phosphonates. Animal behavior in the circular arena was recorded for 3 min as described before [82]. The estimated parameters (duration and number of grooming acts, duration of freezing, number of defecation acts, number of rearing acts, number of crossings for each type of lines (outer circle, inner circle, and sector borders)) served as indicators of anxiety level, locomotor, or exploratory activity according to established views [100].

ECG was recorded using non-invasive electrode placement as previously described [82]. The following parameters were assessed through ECG within the registration time of 3 min: the average interval between the heartbeats (R-R interval, ms), the standard deviation of the average R-R interval values (SD, ms), the range of R-R interval values, i.e., the difference between the maximal and minimal values (dX, ms), the root mean square of successive differences in R-R intervals (RMSSD, ms), and stress index (SI, arbitrary units). SI is calculated using the formula SI = AMo/(2 × Mo) × dX, where Mo is the mode of R-R intervals, i.e., the most frequent R-R value on the histogram; AMo is the mode amplitude, i.e., the incidence count of the most frequent values [101]. Among these ECG parameters, SI is a measure of animal stress, opposed to RMSSD manifesting adaptability of the organism to stress, that is related to the heart rate variability parameters SD and dX [19,101,102]. 

### 4.4. Metabolite and Enzyme Activity Assays

Methanol/acetic acid extracts of rat cerebral cortex were prepared and used for the quantification of amino acids and related compounds in the L-8800 amino acid analyser (Hitachi, Tokyo, Japan) as described previously [103]. Briefly, 0.5 g of brain tissue was homogenized in 4 mL ice-cold methanol, with the resulting homogenate diluted with 0.2% acetic acid at a 1:1.5 ratio. After a 30 min incubation, the suspension was centrifuged and clear supernatants were stored at −70 °C until analysis. The 150 µL sample aliquots were loaded onto the ion-exchange column 2622SC-PF (Hitachi, Ltd., P/N N 855-4507, 4.6 × 60 mm) and eluted by a step-wise gradient of lithium acetate buffers (Wako Pure Chemical Corporation, Osaka, Japan; the pH values of the buffers were 2.8 (PF1), 3.7 (PF2), 3.6 (PF3) and 4.1 (PF4)) at a flow rate of 0.4 mL/min and temperature gradient of 30–70 °C. Post-column derivatization (flow rate of 0.35 mL/min at 136 °C) was performed using the Ninhydrine colouring solution kit (Wako Pure Chemical Industries, Osaka, Japan). Coloured products were detected by absorption at 570 and 440 nm. Data were processed using MultiChrom for Windows software (Ampersand Ltd., Moscow, Russia).

NAD^+^ level was measured using a fluorometric assay employing recombinant formate dehydrogenase [104]. A volume of 10–50 µL of the methanol/acetic acid extracts was assayed in 0.1 M sodium phosphate buffer, pH = 7.0, containing 0.6 M formate and 0.02–0.06 units of recombinant formate dehydrogenase per assay in 200 µL microplate well using CLARIOstar Plus microplate reader (BMG Labtech, Ortenberg, Germany).

Oxidized glutathione was quantified using a fluorometric assay employing a reaction with *ortho*-phthalaldehyde in an alkaline medium [105,106], modified for the microplate format. Extracts of 25 µL were mixed with 10 µL of 40 mM N-ethylmaleimide, incubated for 30 min at room temperature, and then diluted with 415 µL of 0.1 M NaOH. A volume of 10–50 µL of the resulting extracts was further diluted with 0.1 M NaOH up to 197 µL in a microplate well. After the measurement of background fluorescence, 3 µL of 25 mg/mL *ortho*-phthalaldehyde in methanol were added to each well. The fluorescence intensity, measured 30 min after *ortho*-phthalaldehyde addition with the CLARIOstar Plus microplate reader, was used to calculate the content of oxidized glutathione per well and then per g of tissue fresh weight. The calibration curve employed 5–250 pmol of oxidized glutathione per well.

Enzyme activities were measured in cerebral cortex homogenates as described previously [51]. Briefly, 0.5 g of brain tissue was homogenized in 1.25 mL of homogenization buffer (50 mM MOPS, pH = 7.0, containing 2.7 mM EDTA, 20% glycerol and protease inhibitors: 0,2 mM AEBSF, 0.16 µM aprotinin, 3.33 µM bestatin, 3 µM E-64, 2 µM leupeptin, and 1.4 µM pepstatin A) using a ULTRA-TURRAX^®^ T-10 Basic disperser (IKA, Staufen, Germany). The homogenates were sonicated in a Bioruptor^®^ (Diagenode, Liege, Belgium), mixed with solubilization buffer (40 mM Tris-HCl, pH = 7.4, containing 600 mM NaCl, 4 mM EDTA, 1% sodium deoxycholate, and 4% NP-40) at a 3:1 ratio, and incubated for at least 30 min before measurement. Activities of glutamate dehydrogenase, malate dehydrogenase, malic enzyme, and OGDH were measured spectrophotometrically by monitoring the absorbance of NAD(P)H at 340 nm. As all substrates and coenzymes in the assays were at saturating concentrations, and SP is known to dissociate from OGDH upon dilution, the assayed maximal activities are proportional to the expression of assayed enzymes but does not reflect the native fluxes through the enzymes, dependent on the in vivo concentrations of the substrates, cofactors, and inhibitors. 

Glutamine synthetase activity was assayed by an endpoint method measuring the absorbance of the side reaction product, γ-gutamyl hydroxamate-Fe^3+^ complex in acidic condition at 540 nm [51,107].

Pyruvate dehydrogenase activity was measured using a coupled reaction with iodonitrotetrazolium, as described in [108], with several modifications, including omission of dithiothreitol from the reaction medium, suggested in [109]. The final assay medium comprised of 50 mM potassium phosphate buffer, 5 mM L-carnitine, 2.5 mM NAD^+^, 0.2 mM ThDP, 0.1 mM coenzyme A, 1 mM MgCl_2_, 1 mg/mL bovine serum albumin, 0.6 mM iodonitrotetrazolium chloride, 6.5 µM phenazine methosulfate, and 5 mM pyruvate, pH = 7.5. The reaction was started by the addition of 5 µL of homogenate, diluted 5 times in the homogenization buffer. The slope in the absorbance change at 500 nm was corrected for the slope in the reaction medium omitting pyruvate, and molar absorption of iodonitrotetrazolium formazan (ε = 12,400 M^−1^ × cm^−1^ [110]) was used to calculate the activity.

The levels of all low-molecular-weight metabolites were calculated in µmoles per gram of tissue fresh weight. The assayed enzyme activities were expressed as µmoles of substrate consumed per minute per gram of tissue fresh weight.

### 4.5. Statistics and Data Analysis

Data are presented as mean ± standard error of the mean for each experimental group. Experimental groups were compared using one-way ANOVA with Tukey’s post-hoc test, employed in GraphPad Prism 9.0 (GraphPad Software Inc., La Jolla, USA). The differences with *p* ≤ 0.05 were considered significant. Outliers (indicated as hollow points in graphs) were identified according to the iterative Grubb’s test (alpha = 0.01) and excluded from statistical analysis. The number of animals in experimental groups (n) is indicated including outliers.

According to the Shapiro-Wilk test, some parameters (e.g., physiological indicators with discrete scale) were not normally distributed. Thus, Spearman’s correlation coefficients were calculated for each pair of the parameters. Correlation matrices were prepared using RStudio 1.4 (RStudio, PBC, Boston, MA, USA) and Adobe Illustrator 24.2 (Adobe, Inc., San Jose, CA, USA). The correlations with *p* ≤ 0.05 were considered significant.

## 5. Conclusions

Increasing inhibition of OGDH switches the brain metabolism to a state with decreased glutathione redox potential, preceded by a transient compensatory response of the brain metabolic network. The compensatory response activated at a low level of OGDHC inhibition supports stabilization of the animal ECG and behavioral parameters. The perturbed redox state induced by strong inhibition of OGDH impairs central mechanisms of animal stress adaptation. The impairment is obvious from the shifted parameters of the heart rate variability, increased anxiety, and reduced locomotion. Our findings on the physiological impact of decreased function of the brain OGDHC suggest that patients with different neurogenerative and psychiatric disorders, associated with decreased function of the brain OGDHC, may benefit from the OGDH upregulation, e.g., through administration of the physiological activator of OGDH, thiamine, or its pharmacological forms.

## Data Availability

Data is contained within the article.
